# Fluticasone impact on airway dendritic cells in smokers: a randomized controlled trial

**DOI:** 10.1186/1465-9921-14-114

**Published:** 2013-10-29

**Authors:** Marek Lommatzsch, Ulrike Kraeft, Laura Troebs, Katharina Garbe, Andrea Bier, Paul Stoll, Sebastian Klammt, Michael Kuepper, Kai Bratke, Johann Christian Virchow

**Affiliations:** 1 Department of Pneumology, University of Rostock, Rostock, Germany; 2 Department of Tropical Medicine and Infectious Diseases, University of Rostock, Rostock, Germany; 3 Abteilung für Pneumologie und Internistische Intensivmedizin, Zentrum für Innere Medizin, Universität Rostock, Ernst-Heydemann-Str. 6, 18057 Rostock, Germany

**Keywords:** Smoking, Dendritic cells, Inhaled corticosteroids

## Abstract

**Background:**

Myeloid Dendritic cells are key drivers of inflammation in smoke-related lung diseases, whereas plasmacytoid DCs play a crucial role in the defense against infections. Effects of inhaled corticosteroids (ICS) on airway DCs in smokers are unknown.

**Methods:**

In this randomized, double-blind, placebo-controlled clinical trial, 45 active cigarette smokers inhaled placebo, fluticasone or fluticasone plus salmeterol twice daily for 4 weeks. Bronchoalveolar lavage fluid DCs were analyzed using four-color flow cytometry before and after the inhalation period. In addition, fluticasone effects were tested on T-cell proliferation in co-cultures with blood myeloid DCs from smokers.

**Results:**

Inhalation of fluticasone plus salmeterol, but not fluticasone alone or placebo, reduced endobronchial concentrations of myeloid DCs (median decrease: 24%), macrophages (median decrease: 26%) and neutrophils (median decrease: 76%). In contrast, fluticasone reduced plasmacytoid DC concentrations independently of salmeterol. There were no changes in the expression of function-associated surface molecules on myeloid DC (such as CD1a, Langerin, BDCA-1, CD83 or CCR5) in all groups after treatment. Fluticasone (either alone or in combination with salmeterol) suppressed T-cell proliferation in co-cultures with blood myeloid DCs from smokers.

**Conclusions:**

Resistance to ICS monotherapy in smokers might in part be due to lacking effects on airway myeloid DCs, whereas the increased risk for infections during ICS therapy could be attributable to a reduction in plasmacytoid DCs. Combination therapy of fluticasone with salmeterol is associated with a reduction in airway myeloid DCs, but also airway macrophages and neutrophils.

**Trial registration:**

Registered at ClinicalTrials.gov (identifier: NCT00908362) and the European Clinical Trial Database, EudraCT (identifier: 2009-009459-40).

## Background

Smoking is the most prominent risk factor for the development of inflammatory lung diseases such as chronic obstructive pulmonary disease (COPD), Pulmonary Langerhans Cell Histiocytosis (PLCH) or Respiratory Bronchiolitis Interstitial Lung Disease (RB-ILD) [1]. In patients with asthma, smoking reduces asthma control and the therapeutic response to inhaled contricosteroids (ICS) [2]. There is growing evidence that a pathologic accumulation and activation of Dendritic cells (DCs) within the lung plays a central pathogenetic role in these diseases [3-5]. Airway DCs are professional antigen presenting cells which control pulmonary immune responses by regulating the expansion of specific pro-inflammatory or anti-inflammatory T-cell subsets [3]. Under physiological conditions, DCs migrate to the draining lymph nodes in order to present antigenic information to lymphocytes. However, mainly under pathological conditions, DCs can also present antigens locally to lymphocytes within the lung parenchyma [6]. DCs are subdivided into myeloid DCs (mDCs) and plasmacytoid DCs (pDCs). The mDCs initiate and promote inflammation in various diseases of the airways, including asthma and respiratory infections [7,8]. The functional role of pDCs in humans is less clear. Current concepts postulate that pDCs might have anti-inflammatory properties and that pDCs play a crucial role in the defense against infections [7,8].

In the airways of smokers, a characteristic expansion of Langerhans cells (a subset of mDCs) can be observed [9]. In addition, airway mDCs of smokers are characterised by changes in function-associated surface molecules, such as an increased expression of the co-stimulatory molecules CD80 and CD86, and an increased expression of antigen recognition receptors such as BDCA-1 (CD1c) and MMR (Macrophage Mannose Receptor) [9]. Smoke-related lung diseases such as COPD and PLCH are accompanied by a local accumulation of DCs in the lungs which exceeds DC numbers in smoking controls [1,10-12]. In addition, phenotypic characteristics of DCs appear to differ between “healthy” smokers and patients with smoke-related lung diseases [13]. Although there is a substantial body of evidence that corticosteroids influence airway DCs in patients with allergic asthma and allergic rhinitis [14-16], there is currently no information on the effect of ICS on airway DCs in cigarette smokers with or without smoke-related lung diseases. Therefore, it was the aim of this trial to investigate the influence of ICS inhalation on airway DCs in smokers for the first time. Participant selection was limited to smokers with normal spirometry, because the local ethics committee of Rostock (Germany) stated that it is ethically inappropriate to treat patients with COPD (of any severity) with placebo only. Using an established flow cytometric method to analyze DCs in human bronchoalveolar lavage (BAL) fluid [17-19], we measured airway DCs in smokers prior to and after 4 weeks of inhalation of fluticasone, and compared the findings with the effects of placebo treatment and of combination therapy (fluticasone plus salmeterol), in a randomized, double-blind, placebo-controlled clinical trial.

## Methods

### Study approval and endpoints

The study was approved by the ethics committee of Rostock (Germany), the german regulatory authority (Bundesinstitut für Arzneimittel und Medizinprodukte, BfArM, http://www.bfarm.de), the European Medicines Agency (EMA, http://www.ema.europa.eu, registration number EUDRACT 2009-009459-40), and registered with http://www.ClinicalTrials.gov (NCT00908362). All participants gave their written informed consent. Primary endpoint were DC concentrations in BAL fluid, secondary endpoints were mDC surface molecules in BAL fluid after treatment with the study drug. The study was supported by GlaxoSmithKline (GSK, Brentford, UK).

### Participants

Participants were recruited in Rostock (Germany), using these inclusion criteria: 1. men between 30 and 60 years, 2. current smoking of at least 10 cigarettes per day and no intention to stop smoking despite counseling about adverse effects of smoking and methods to quit, 3. smoking history of at least 15 years. Exclusion criteria were: 1. any history of chronic diseases (except arterial hypertension), 2. any regular medication (except medication for arterial hypertension), 3. respiratory tract infections within the last week prior to study inclusion, 4. an oxygen saturation below 90%, 5. a forced expiratory volume in the first second (FEV_1_) < 80% of the predicted value.

### Study design and medication

**Figure 1:**
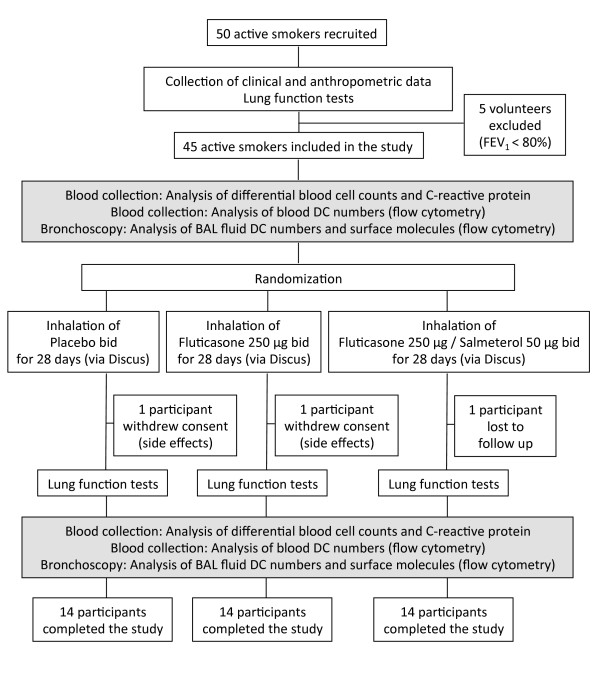
**Study flow diagram.** Fourty-five smokers were included into the study, based on lung function, smoking history and clinical data. Participants were randomized to inhale Placebo, Fluticasone or the combination of Fluticasone and Salmeterol twice daily for 4 weeks. Prior to the first inhalation and directly after the last inhalation of the study drug, participants underwent body plethysmography, blood collection and bronchoscopy (with bronchoalveolar lavage).

The study flow diagram is shown in Figure 1. Lung function was measured using body plethysmography (Masterscreen, Carefusion, Hoechberg, Germany). Following study inclusion, blood was collected (for the analysis of blood cell counts, C-reactive protein and blood DCs) and the first bronchoscopy was performed. Afterwards, participants obtained a blinded Discus® (labeled with the participant’s number according to the randomization list). The inhalation technique was demonstrated and checked. Participants were instructed to use the device twice daily for 28 days. The devices (provided by GlaxoSmithKline, GSK, Brentford, UK) contained one of the three study medications: placebo, fluticasone (250 μg per dose) or fluticasone (250 μg per dose) plus salmeterol (50 μg per dose). Participants and investigators were unaware of the study medication. Adherence was measured using dose counters in the devices. Directly after the inhalation period, lung function testing, blood collection and the second bronchoscopy was performed. There was a follow-up over 14 days after the second bronchoscopy which mainly included clinical monitoring of the participants.

### Bronchoalveolar lavage and flow cytometry

**Figure 2:**
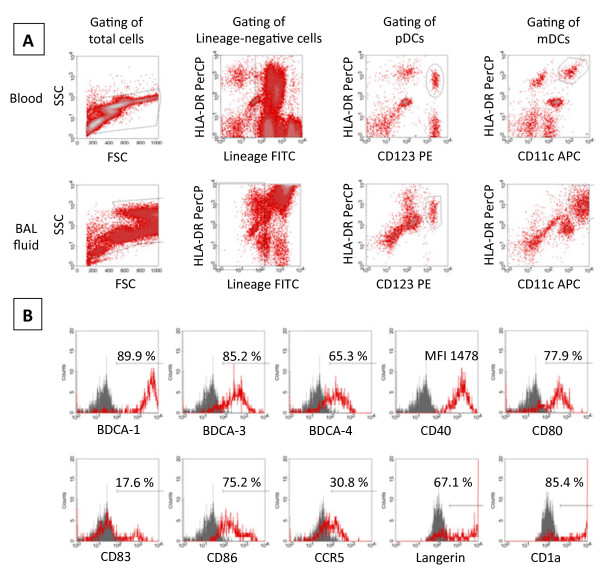
**Gating strategy and mDC surface molecule analysis. A** (gating strategy): Cells were identified in a FSC/SSC-Plot (first column), and Lineage negative/dim (linneg/dim) cells gated (second column). Among linneg/dim cells, plasmacytoid dendritic cells (pDCs) were identified as CD123 and HLA-DR positive cells (third column), and myeloid dendritic cells (mDCs) as CD11c and HLA-DR positive cells (fourth column). **B** (analysis of surface molecule expression): Surface molecules on mDCs were quantified in histogram-plots using isotype control antibodies to discriminate between specific (red) and non-specific (grey) antibody staining. The expression of surface molecules was either calculated as the percentage of positive mDCs or as the strength of expression (MFI: mean fluorescence index). The results of one representative participant are shown.

BAL was performed in the middle lobe using flexible bronchoscopes (Olympus, Hamburg, Germany) with 100 ml of sterile saline. BAL fluid cells were isolated and differential cell counts determined as described [17-19]. DCs in BAL fluid and blood were analyzed using four-colour flow cytometry (see antibody panel in Additional file 1: Table S1) as described [17-19]. The concentration of blood DCs was calculated using the following formula: (*%*DCs/100) × blood leukocyte concentration. The concentration of BALF DCs was calculated using the following formula: (*%*DCs/100) × BALF cell concentration. At least 150000 events were measured. The gating strategy and histogram plots for DC surface molecule analysis are shown in Figure 2.

### Isolation of cells for cell culture experiments

Blood mDCs were isolated from asymptomatic smokers using a commercial CD1c (BDCA-1)+ Dendritic Cell Isolation Kit (Miltenyi Biotec, Bergisch Gladbach, Germany) as described [20]. In all experiments, purity of blood mDCs was > 90%. Autologous blood CD4+ T-cells were isolated using the commercial CD4+ T cell Isolation Kit II (Miltenyi Biotec, Bergisch Gladbach, Germany) as described [20].

### Cell cultures and T-cell proliferation assays

Co-cultures of mDCs and autologous T-cells were performed as described [20]. Briefly, isolated CD4+T cells were resuspended in PBS containing 0.1% bovine serum albumin (1 million cells/ml) and labelled at 37°C for 10 min with 1 μM CFSE (CarboxyFluorescein diacetate Succinimidyl Ester)(Invitrogen, Karlsruhe, Germany). Labelling was stopped with ice-cold RPMI 1640 followed by an incubation on ice for 5 min and three washing steps with medium. CFSE-labelled CD4+T cells (1 million cells/ml) were co-cultured with blood mDCs at a 5:1 ratio in RPMI 1640 containing 10% fetal calf serum for 5 days, and stimulated with LPS (10 μg/ml, Invitrogen, San Diego, CA, USA). LPS-stimulated cell cultures were incubated with medium alone, Fluticasone (10^-7^ M) or Fluticasone (10^-7^ M) plus Salmeterol (10^-7^ M)(GSK, Brentford, UK) as described [21]. After 5 days of culture, cells were harvested, washed with PBS (containing 2% fetal calf serum) and stained with anti-human CD3 (label: APC) for 20 min. After washing, cells were resuspended in PBS containing propidium iodide (PI, 2 μg/ml) to label dead cells. T-cell proliferation was quantified by measuring CFSE dilution of the CD3+PI- cells, using a FACSCalibur™ with BD CellQuest Pro™ Software (BD Biosciences, Heidelberg, Germany).

### Statistical analysis

Data were analysed using SPSS (Chicago, IL, USA). Parameters were calculated as medians (minimum - maximum). The comparison of parameters between the time points A (prior to the first inhalation) and B (directly after the inhalation period) was performed using the Wilcoxon test for related samples. T-cell proliferation rates in cell cultures were compared using the Mann–Whitney U test. Probability values of p < 0.05 were regarded as significant.

## Results

### Characteristics of the participants

Fourty-five male smokers were included in the study based on the inclusion and exclusion criteria. In each group, there was one drop out during the 4-week inhalation period: two participants withdrew consent due to mild side effects, one participant was lost to follow-up (Figure 1). The characteristics of the participants in the 3 groups who completed the study are detailed in Table 1.

**Table 1:** Characteristics of the participants

**Parameter**	**Placebo**	**Fluticasone**	**Fluticasone/Salmeterol**
**Participants (who completed the study)**	14	14	14
**Age (years)**	45 (30 - 61)	35 (30 - 55)	44 (34 - 60)
**Body height (cm)**	179 (166 - 188)	180 (168 - 190)	179 (164 - 183)
**Body weight (kg)**	76 (60 - 103)	83 (60 - 108)	79 (60 - 120)
**Pack Years**	30 (8 - 70)	23 (11 - 62)	25 (8 - 78)
**Cigarettes / Day**	17 (10 - 30)	19 (12 - 30)	17 (10 - 50)

Shown are median values (minimum – maximum) of the age, anthropometric parameters and smoking history of the participants who completed the study.

Analysis of the Discus® dose counters after the 4-week inhalation period revealed that the participants had inhaled a median of 55 (98%) of the 56 doses, with no significant differences between the 3 groups. Lung function parameters of the participants before and after the 4-week inhalation period are specified in Additional file 1: Table S2. Differential blood counts and concentrations of C-reactive protein (CRP) of the participants before and after the 4-week inhalation period are specified in Additional file 1: Table S3. In all 3 groups, there were no significant differences in lung function parameters, differential blood cell counts or CRP concentrations between the two time points.

### Differential cell counts in BAL fluid

The recovered BAL fluid volumes, and the total and differential leukocyte counts in BAL fluid at the time points A and B are detailed in Table 2. The BAL fluid recovery did not differ between the two time points in all 3 groups. After 4 weeks of inhalation, there was a significant decrease in total cell counts, macrophage counts and neutrophil counts in the group treated with fluticasone plus salmeterol, but not in the group treated with fluticasone alone or placebo (Table 2). In contrast, none of the study medications had a significant impact on lymphocyte or eosinophil counts in BAL fluid (Table 2).

**Table 2:** BAL fluid analysis

	**Placebo**		**Fluticasone**		**Fluticasone/Salmeterol**	
**Time point**	**A**	**B**	**A**	**B**	**A**	**B**
**n**	**14**	**14**	**14**	**14**	**14**	**14**
**Recovery (ml BAL fluid)**	62 (45 - 67)	64 (48 - 71)	63 (56–70)	65 (37–72)	62 (55–65)	62 (54–70)
**Total cells (10**^**3**^**/ml BAL fluid)**	121 (17–487)	190 (35–469)	81 (38–284)	93 (6–217)	125 (36–409)	97* (25–339)
**Macrophages (10**^**3**^**/ml BAL fluid)**	114 (15–447)	181 (34–459)	75 (32–263)	86 (5–205)	119 (32–397)	88* (22–315)
**Lymphocytes (10**^**3**^**/ml BAL fluid)**	7.2 (0.5 - 34.1)	5.6 (0.7 - 28.2)	2.7 (0.6 - 13.1)	4.1 (0.6 - 7.8)	3.0 (1.0 - 39.1)	3.2 (0.7 - 14.2)
**Neutrophils (10**^**3**^**/ml BAL fluid)**	2.1 (0.3 - 12.0)	1.4 (0.0 - 5.0)	1.6 (0.6 - 5.1)	1.2 (0.2 - 6.1)	2.9 (0.1 - 5.4)	0.7* (0.0 - 6.1)
**Eosinophils (10**^**3**^**/ml BAL fluid)**	0.4 (0.0 - 5.1)	0.3 (0.0 - 4.2)	0.5 (0.0 - 2.8)	0.2 (0.0 - 1.2)	0.1 (0.0 - 3.0)	0.2 (0.0 - 3.4)

Shown are median values (minimum – maximum) of the recovery of BAL fluid, the total concentration of BAL fluid cells and the concentrations of macrophages, lymphocytes, neutrophils and eosinophils in BAL fluid of the participants (median values, minimum – maximum) in the 3 study arms (Placebo, Fluticasone, Fluticasone/Salmeterol), at the time points A (prior to treatment) and B (directly after treatment). Significant differences between the time points (p < 0.05) are marked with an asterisk (*).

### DC concentrations in blood and BAL fluid

**Figure 3:**
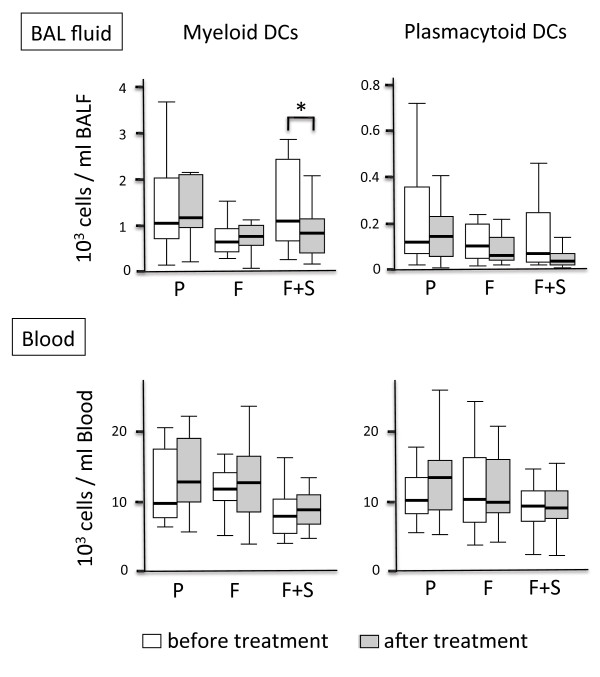
**DCs in BAL fluid and blood: comparison of the study arms.** Shown are the concentrations of myeloid and plasmacytoid DCs in BAL fluid (upper panel) and peripheral blood (lower panel) prior to the first inhalation of the study drug (open boxes) and directly after 4 weeks of inhalation (grey boxes). Boxplots display the median (line within the box), interquartil range (edges of the box) and extremes (vertical lines). Outliers (all cases more distant than 1.5 interquartil ranges from the upper or lower quartil) were omitted in the graphs. Significant differences between the two time points (p < 0.05) are marked with an asterisk. *Abbreviations denote:* Group (n = 14) assigned to Placebo (P), group (n = 14) assigned to Fluticasone (F), group (n = 14) assigned to Fluticasone and Salmeterol (F+S).

**Figure 4:**
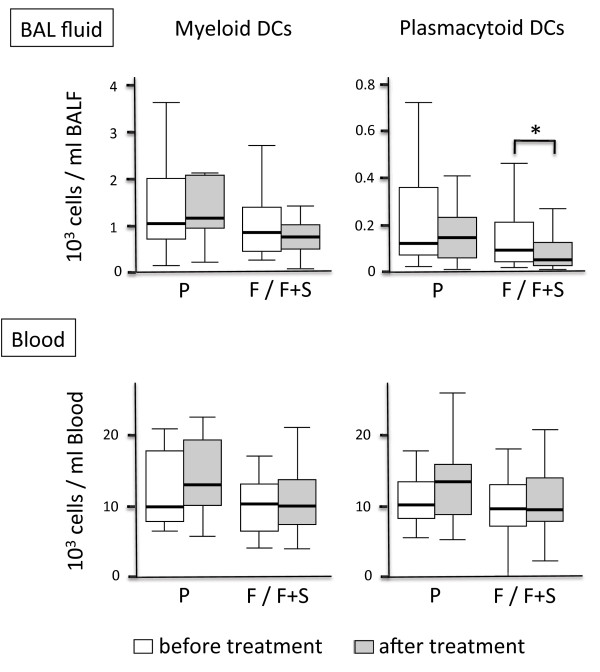
**DCs in BAL fluid and blood: combined analysis of fluticasone groups.** Shown are the concentrations of myeloid and plasmacytoid DCs in BAL fluid (upper panel) and peripheral blood (lower panel) prior to the first inhalation of the study drug (open boxes) and directly after 4 weeks of inhalation (grey boxes). Boxplots display the median (line within the box), interquartil range (edges of the box) and extremes (vertical lines). Outliers (all cases more distant than 1.5 interquartil ranges from the upper or lower quartil) were omitted in the graphs. Significant differences between the two time points (p < 0.05) are marked with an asterisk. *Abbreviations denote:* Group (n = 14) assigned to Placebo (P), combined analysis (n = 28) of the groups assigned to Fluticasone or Fluticasone and Salmeterol (F / F+S).

The concentrations of mDCs and pDCs in BAL fluid and in blood at the time points A and B are shown in Figure 3. In all 3 groups, blood DC concentrations were unaffected after the 4-week inhalation period. Myeloid DCs in BAL fluid significantly decreased only in the group treated with fluticasone plus salmeterol, but not in the group treated with fluticasone alone or placebo (Figure 3). Inhalation of placebo did not alter pDC concentrations in BAL fluid. There was a decrease in pDC concentrations in the groups treated with fluticasone (p = 0.12) and fluticasone/salmeterol (p = 0.13), but this decrease was not significant (Figure 3). Combined analysis of all fluticasone treated participants (fluticasone group and fluticasone/salmeterol group, n = 28 participants) revealed that inhalation of fluticasone significantly reduced pDCs in the BAL fluid of smokers, independently of salmeterol (Figure 4).

### Surface molecule expression on mDCs in BAL fluid

The expression of mDC surface molecules BDCA-1, BDCA-3, BDCA-4, CD40, CD80, CD83, CD86, CCR5, Langerin and CD1a in BAL fluid is detailed in the (Additional file 1: Table S4). There were no significant changes in the proportions of cells (% positive) expressing the surface markers between the time points A and B. In case of CD40, there was no significant difference in the level of expression (MFI) between the time points A and B (Additional file 1: Table S4).

### Fluticasone effects on T-cell proliferation in mDC-T-cell co-cultures

**Figure 5:**
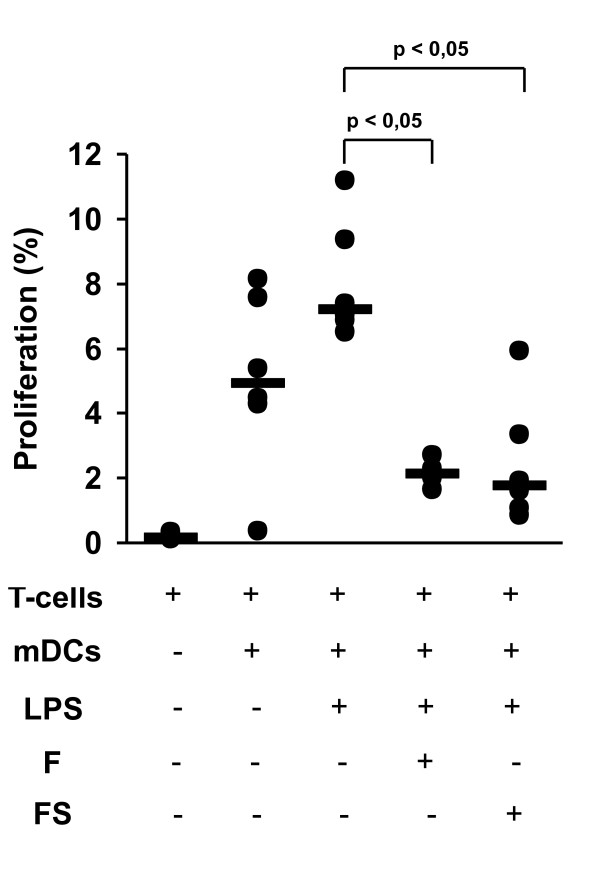
**T-cell proliferation in DC-T-cell co-cultures.** Autologous peripheral blood CD4+T cells were cultured alone (first column) or co-cultured with blood mDCs (second to fifth column) at a 5:1 ratio for 5 days. LPS-stimulated co-cultures (third to fifth column) were incubated with medium alone (third column), Fluticasone (F; fourth column) or Fluticasone plus Salmeterol (F+S; fifth column). The graph shows the T-cell proliferation rate after 5 days of culture. Significant differences (p < 0.05) are marked.

In order to test whether fluticasone impacts on the interaction between mDCs and T-cells of cigarette smokers, mDCs isolated from peripheral blood of asymptomatic smokers were co-cultured with autologous peripheral blood T-cells and stimulated with 1 μg/ml LPS (n = 6 active cigarette smokers; n = 3 experiments per smoker) (Figure 5). LPS-stimulated cell cultures were incubated with fluticasone (10^-7^ M) or with fluticasone (10^-7^) plus salmeterol (10^-7^ M). Both incubation with fluticasone and with fluticasone plus salmeterol strongly reduced T-cell proliferation in LPS-stimulated mDC-T-cell co-cultures (Figure 5).

## Discussion

DCs are crucial players in pulmonary diseases caused by tobacco smoke, but knowledge on the pharmacologic modulation of airway DCs in smokers is sparse. This study demonstrates for the first time that inhalation of the ICS fluticasone reduces pDC numbers in the airways of cigarette smokers. In addition, it demonstrates that only a combination therapy of fluticasone with the long-acting beta-agonist (LABA) salmeterol reduces airway mDCs in smokers. Notably, neither inhalation of fluticasone alone nor a combination therapy modulated the phenotype of airway DCs. Thus, our study adds important new evidence to the ongoing discussion on the role of ICS in the treatment of smokers.

Corticosteroids can influence the number, phenotype and function of human mDCs [22,23]. Several studies have demonstrated that ICS reduce mDCs in the airways of healthy subjects and of patients with allergic asthma [14,15,24]. Our study is the first to investigate ICS effects on airway DCs in smokers. In contrast to the findings in healthy subjects and patients with asthma [14,15,24], there was no reduction in endobronchial mDCs after fluticasone monotherapy. In addition, there were no changes in the expression of function-associated mDC surface molecules. There are two hypotheses to explain these findings. On the one hand, airway mDCs of smokers might be ICS resistant, according to the concept that smokers exhibit a relative corticosteroid resistance compared to non-smokers [25]. On the other hand, the 4-week inhalation period might be too short or the ICS dose chosen too low to cause effects on endobronchial mDCs in smokers. However, a previous report using the same fluticasone dose as in our study demonstrated a reduction in airway mDCs in patients with asthma and in healthy controls in a comparable time period [14]. Therefore, the lacking effects of fluticasone monotherapy on airway mDCs in smokers might be rather due to a resistance to the drug than due to the dose of fluticasone or the duration of the treatment.

An abnormal response of the innate and adaptive immune system to noxious particles and gases is the key pathogenetic feature of chronic obstructive pulmonary disease (COPD) [3]. Only combined therapy of ICS with LABA, but not ICS monotherapy, is an effective treatment option for COPD. This clinical experience is supported by molecular evidence suggesting that the crosstalk between ICS and LABA potentiates the anti-inflammatory effect of ICS [26]. Accordingly, ICS resistance of patients with COPD can be reversed by inhalation of a LABA [27-29]. Our study confirms these data by showing that only smokers treated with fluticasone plus salmeterol, but not smokers treated with fluticasone alone, displayed a decrease in endobronchial macrophages and neutrophils. In addition, we demonstrate for the first time that only inhalation of fluticasone plus salmeterol reduces endobronchial mDCs in smokers. Myeloid DCs have been postulated to be key drivers of inflammation in smoke-related lung diseases [3,30]. Thus, we hypothesize that the mDC reduction induced by the inhalation of fluticasone plus salmeterol might lead to a sustainable reduction of inflammation in the airways of cigarette smokers. It has been postulated that specific mDC subsets might play a major role in the pathogenesis of smoke-related lung diseases [31]. However, we did not observe a preferential decrease in one specific subset (such as CD1a+ mDCs or BDCA-1+ mDCs) following combination therapy. Thus, combination therapy might not only reduce those mDC subsets which induce the pathology of smoke-related lung diseases, but also other mDC subsets which might be beneficial. In addition, it has to be noted that mDCs also play a role in the protection against infections [8]. A reduction of mDCs in the airways may, therefore, predispose for infections. Thus, further studies are needed to clarify the precise role of mDC subsets in smoke-related lung diseases.

In additional DC-T-cell co-culture experiments using DCs from peripheral blood of active cigarette smokers we observed that the supression of DC-induced T-cell proliferation by fluticasone is not dependent on the presence of salmeterol. Thus, it appears that some aspects of DC pathophysiology can be influenced by ICS alone (such as the induction of T-cell proliferation), while others can only be influenced by a combination of ICS plus LABA (such as the number of mDCs in the airways).

Another effect of fluticasone independent of salmeterol co-medication was the reduction of endobronchial pDCs after 4 weeks of treatment. Plasmacytoid DCs are thought to have beneficial effects in chronic inflammatory airway diseases due to anti-inflammatory properties and due to their crucial role in the defense against infections [7,8]. However, pDCs are very sensitive to corticosteroids. Corticosteroids such as prednisolone or dexamethasone inhibit the differentiation and induce apoptosis of human pDCs [32,33]. It has been suggested that a corticosteroid-induced reduction of endobronchial pDCs might be related to the increased risk of recurrent pneumonias [18]. An increased risk of pneumonia has also been identified as a side effect of ICS therapy in patients with COPD [34], although the underlying mechanisms for this clinical observation are still unknown. The reduction of airway pDCs by fluticasone observed in our study might be one contributing mechanism. However, studies in patients with COPD are needed to further clarify this issue.

A limitation of the study is the fact that only smokers with normal spirometry were included in the trial. Therefore, the relevance of the findings of this study for patients with COPD or other smoking-related lung diseases remains open. It was necessary to include a placebo group in this trial in order to exclude artifacts due to the endoscopic intervention and to exclude a possible physiologic variation of DC concentrations in the airways of smokers over the chosen time period. The scientific decision to include a placebo group led to the statement of the ethics committee that smokers with COPD (of any severity) must be excluded from the study because it was felt that treatment of patients with COPD with placebo only was ethically inappropriate. There is an ongoing debate about the design of clinical trials in the era of new COPD treatment options. The availability of potent drugs for this devastating disease makes it ethically impossible to perform controlled trials including a group of patients treated with placebo only. This results in the difficulty to distinguish between direct effects of the studied compound on the disease and complex drug-drug interactions with baseline therapy.

## Conclusions

This randomized, double-blind, placebo-controlled clinical trial demonstrates for the first time that fluticasone reduces airway mDCs of smokers only in combination with salmeterol, whereas the effect of fluticasone on airway pDCs is independent of salmeterol. These findings will help to better understand ICS effects on inflammatory airway diseases of smokers.

## Abbreviations

BAL: Bronchoalveolar Lavage; BDCA: Blood dendritic cell antigen; CCR5: C-C Chemokine receptor 5; CD: Cluster of differentiation; COPD: Chronic obstructive pulmonary disease; DC: Dendritic cell; FACS: Fluorescence activated cell sorter; FEV1: Forced expiratory volume in the first second; ICS: Inhaled corticosteroid; LABA: Long-acting beta-agonist; mDC: Myeloid dendritic cell; MMR: Macrophage mannose receptor; pDC: Plasmacytoid dendritic cell.

## Competing interests

UK, LT, PS AB, SK, KG, MK, KB have no conflicts of interest. ML and JCV served in advisory boards and/or received lecture fees from the following companies: Astra Zeneca, Boehringer Ingelheim, Chiesi, GSK, Janssen, MSD, Novartis.

## Authors’ contributions

ML and JCV designed and supervised the study, wrote the proposals for the authorities, analyzed the data and wrote the manuscript draft; UK, LT and PS recruited and characterized the participants, and collected the clinical data; AB and ML performed the bronchoscopies; SK designed the study and wrote the proposals for the authorities; KG, MK and KB performed flow cytometric analyses, cell purifications and cell cultures. All authors read and approved the final manuscript.

## Supplementary Material

Additional file 1 **Supplement. Table S1.** Antibodies used for four-colour flow cytometry. **Table S2.** Lung function of the Participants. **Table S3.** Blood parameters of the Participants. **Table S4.** Surface molecule expression on BAL fluid myeloid DCs (mDCs). Click here for file
