# Supplementary material for: Fluticasone impact on airway dendritic cells in smokers: a randomized controlled trial
Source: Respir Res. 2013 Oct 29;14(1):114. doi: 10.1186/1465-9921-14-114 (PMC4176093; doi:10.1186/1465-9921-14-114)
Supplement: Additional file 1 — Supplement. Table S1. Antibodies used for four-colour flow cytometry. Table S2. Lung function of the Participants. Table S3. Blood parameters of the Participants. Table S4. Surface molecule expression on BAL fluid myeloid DCs (mDCs). [file 1465-9921-14-114-S1.doc]

**Fluticasone impact on airway dendritic cells in smokers:**

**a randomized controlled trial**

Marek Lommatzsch, Ulrike Kraeft, Laura Troebs, Katharina Garbe, Andrea Bier,

Paul Stoll, Sebastian Klammt, Michael Kuepper, Kai Bratke, J. Christian Virchow

**SUPPLEMENT**

| **Antibody** | **Label** | **Company** | **Clone** |
| --- | --- | --- | --- |
| Lineage Cocktail | FITC | BD Biosciences | SK7, MφP9, 3G8, SJ25C1, L27, NCAM16.2 |
| Anti-HLA-DR | PerCP | BD Biosciences | L243 |
| Mouse IgG1 | PE | BD Biosciences | X40 |
| Mouse IgG1 | APC | BD Biosciences | X40 |
| Mouse IgG2a | PE | BD Biosciences | X39 |
| Mouse IgG2a | APC | BD Biosciences | X39 |
| Anti-CD1a | PE | Dako | NA1/34 |
| Anti-CD11c | PE | BD Biosciences | S-HCL-3 |
| Anti-CD11c | APC | BD Biosciences | S-HCL-3 |
| Anti-CD40 | APC | Invitrogen | HB14 |
| Anti-CD80 | APC | ImmunoTools | MEM-233 |
| Anti-CD83 | APC | BD Biosciences | HB15e |
| Anti-CD86 | APC | BD Biosciences | BU63 |
| Anti-CD123 | PE | BD Biosciences | 9F5 |
| Anti-CD207/Langerin | PE | Beckman Coulter | DCGM4 |
| Anti-BDCA-1 | APC | Miltenyi Biotec | AD5-8E7 |
| Anti-BDCA-3 | APC | Miltenyi Biotec | AD5-14H12 |
| Anti-BDCA-4 | APC | Miltenyi Biotec | AD5-17F6 |
| Anti-CCR5/CD195 | APC | BD Biosciences | 2D7 |

**Table S1. Antibodies used for four-colour flow cytometry**

*Abbreviations denote:* Blood Dendritic Cell Antigen (BDCA), Fluorescein isothiocyanate (FITC), Phycoerythrin (PE), Allophycocyanin (APC), Peridinin chlorophyll protein (PerCP). The Lineage cocktail contains antibodies against the following antigens: CD3, CD14, CD16, CD19, CD20, CD56.

|  | **Placebo** | | **Fluticasone** | | **Fluticasone / Salmeterol** | |
| --- | --- | --- | --- | --- | --- | --- |
| **Time Point** | A | B | A | B | A | B |
| **n** | 14 | 14 | 14 | 14 | 14 | 14 |
| **IVC** | 96.8  (42.8 ‑ 124) | 98.3  (89.1 ‑ 121.6) | 99.7  (79.2 ‑ 121.8) | 95.7  (72.9 ‑ 118.5) | 91.2  (77.4 ‑ 119.5) | 94  (81.3 ‑ 123.4) |
| **FVC** | 103.3  (88.6 ‑ 121.1) | 100.3  (83.3 ‑ 122.3) | 97.7  (80.3 ‑ 119.6) | 99.3  (79.5 ‑ 114.5) | 97.1  (80.9 ‑ 125.9) | 96.7  (83.5 ‑ 128.7) |
| **FEV_1_** | 93.9  (82.3 ‑ 110.3) | 92.8  (71.7 ‑ 107.1) | 91  (80.2 ‑ 123.6) | 87.6  (80.7 ‑ 116.6) | 90  (80 ‑ 120.5) | 90.9  (81.6 ‑ 123.5) |
| **FEV_1_%VC** | 74.1  (65 ‑ 81.4) | 74.2  (66.2 ‑ 85) | 79.9  (66.2 ‑ 89.8) | 77.7  (65.9 ‑ 86.8) | 76.1  (70.1 ‑ 84.9) | 76.5  (71.2 ‑ 86.6) |
| **MEF_25_** | 47.5  (26.3 ‑ 97.3) | 41.9  (15.9 ‑ 92.8) | 57.4  (16.1 ‑ 137.9) | 46.6  (11.9 ‑ 122.9) | 44.2  (29.3 ‑ 102.3) | 43.5  (33.1 ‑ 128.1) |
| **RV** | 113.2  (65.6 ‑ 147.4) | 107.5  (83.3 ‑ 163.7) | 87.8  (13.5 ‑ 106.9) | 95.4  (11.3 ‑ 151.5) | 92.5  (62.3 ‑ 136.7) | 93.8  (63.1 ‑ 123.8) |
| **TLC** | 103.9  (84.5 ‑ 130.8) | 98.1  (91.7 ‑ 134) | 95.4  (55.7 ‑ 114) | 99.8  (75.2 ‑ 112.3) | 95  (87.8 ‑ 114.6) | 97.5  (84.2 ‑ 123.1) |
| **RV%TLC** | 106.4  (74.9 ‑ 119.9) | 100.7  (78.9 ‑ 121.6) | 89.9  (58.3 ‑ 116.9) | 87.6  (68.7 ‑ 128.3) | 90.6  (59.3 ‑ 125) | 96  (55.8 ‑ 114.4) |

**Table S2 Lung function of the Participants**

Shown are lung function parameters (median values, minimum – maximum) of the participants in the 3 study arms (Placebo, Fluticasone, Fluticasone/Salmeterol), at the time points A (prior to treatment) and B (directly after treatment). *Abbreviations denote:* Inspiratory Vital Capacity (IVC), Forced Vital Capacity (FVC), Forced Expiratory Volume in the first second (FEV_1_), Maximum Expiratory Flow when 75 % of the FVC is exhaled (MEF_25_), Residual Volume (RV), Total Lung Capacity (TLC). There were no significant differences between the time points in any parameter in any group.

|  | **Placebo** | | **Fluticasone** | | **Fluticasone / Salmeterol** | |
| --- | --- | --- | --- | --- | --- | --- |
| **Time**  **Point** | A | B | A | B | A | B |
| **n** | 14 | 14 | 14 | 14 | 14 | 14 |
| **Hemoglobin**  **(mmol / l)** | 9.1  (8 ‑ 9.8) | 9.1  (8.6 ‑ 9.9) | 9.5  (8.1 ‑ 10.5) | 9.3  (5.4 ‑ 10.5) | 9.4  (8.5 ‑ 10.8) | 9.3  (8.6 ‑ 10.2) |
| **Platelets**  **(10^6^ / ml)** | 234  (146 ‑ 338) | 238  (157 ‑ 341) | 211  (176 ‑ 391) | 218  (178 ‑ 312) | 204  (142 ‑ 320) | 216  (147 ‑ 341) |
| **Leukocytes**  **(10^6^ / ml)** | 6.7  (5.4 ‑ 9.8) | 6.3  (4.7 ‑ 13.9) | 7  (3.5 ‑ 9) | 6.7  (4.6 ‑ 16.1) | 7.2  (4.61 ‑ 13.1) | 7.2  (3.74 ‑ 11.5) |
| **Monocytes**  **(%)** | 8.9  (6 ‑ 10.6) | 8.9  (6 ‑ 14) | 9.2  (6.4 ‑ 13) | 8.3  (6.2 ‑ 13.8) | 7.2  (4 ‑ 12.4) | 7.7  (5 ‑ 11) |
| **Lymphocytes**  **(%)** | 32.7  (16.6 ‑ 47.5) | 29.1  (22 ‑ 44.1) | 32.9  (23.3 ‑ 40.4) | 33.3  (25 ‑ 39.7) | 24.9  (10.3 ‑ 37.2) | 27.6  (15 ‑ 38.6) |
| **Neutrophils**  **(%)** | 56.6  (39.1 ‑ 72.6) | 54.8  (41.9 ‑ 69) | 54.7  (47.1 ‑ 65.2) | 54.5  (46.3 ‑ 62) | 64.0  (6 ‑ 81.1) | 61.8  (47.8 ‑ 71) |
| **Eosinophils**  **(%)** | 2.2  (0.4 ‑ 11) | 2.9  (1.1 ‑ 9.9) | 3.2  (1.3 ‑ 4.9) | 2.8  (1.1 ‑ 6.3) | 2.4  (0.4 ‑  7.4) | 2.0  (0.9 ‑ 6.2) |
| **CRP**  **(mg / l)** | 1.3  (1 ‑ 12.3) | 1.6  (1 ‑ 5.3) | 2.0  (1 ‑ 9.4) | 2.0  (1 ‑ 9.5) | 2.0  (1 ‑ 7.58) | 1  (1 ‑ 11.9) |

**Table S3 Blood parameters of the Participants**

Shown are parameters in peripheral blood (median values, minimum – maximum) of the participants in the 3 study arms (Placebo, Fluticasone, Fluticasone/Salmeterol), at the time points A (prior to treatment) and B (directly after treatment). Monocytes, Lymphocytes, Neutrophils and Eosinophils are given in % of all leukocytes in peripheral blood. *Abbreviation denotes:* C-reactive Protein (CRP). There were no significant differences between the time points in any parameter in any group.

|  | **Placebo** | | **Fluticasone** | | **Fluticasone/Salmeterol** | |
| --- | --- | --- | --- | --- | --- | --- |
| Time Point | A | B | A | B | A | B |
| n | 14 | 14 | 14 | 14 | 14 | 14 |
| **mDC** |  | | | | | |
| BDCA-1  [%] | 89  (58 - 94) | 89  (60 - 94) | 88  (59 - 91) | 85  (71 - 94) | 89  (67 - 94) | 88  (46 - 94) |
| BDCA-3  [%] | 73  (38 - 91) | 74  (42 - 90) | 71  (44 - 92) | 68  (43 - 91) | 63  (24 - 90) | 56  (29 -78) |
| BDCA-4  [%] | 51  (21 - 89) | 46  (29 -80) | 43  (11 - 69) | 51  (10 - 79) | 47  (16 - 79) | 41  (19 - 81) |
| CD40  [MFI] | 1620  (1189 - 3091) | 1733  (1238 - 3005) | 1676  (865 - 2482) | 1887  (1149 - 2430) | 1603  (1173 - 2832) | 1382  (919 - 3289) |
| CD80  [%] | 73  (44 - 85) | 65  (36 - 80) | 63  (35 - 76) | 63  (42 - 79) | 60  (43 - 81) | 58  (39 - 84) |
| CD83  [%] | 17  (6 - 29) | 14  (6 - 24) | 13  (9 - 23) | 14  (5 - 32) | 13  (8 - 31) | 12  (8 - 22) |
| CD86  [%] | 75  (54 - 96) | 68  (53 - 92) | 61  (39 - 94) | 65  (40 - 91) | 68  (30 - 88) | 59  (37 - 93) |
| CCR5  [%] | 26  (6 - 56) | 23  (3 - 48) | 13  (0 - 61) | 15  (2 - 45) | 17  (1 - 44) | 17  (0 - 44) |
| Langerin  [%] | 67  (33 - 76) | 64  (33 - 82) | 61  (44 - 75) | 62  (41 - 73) | 61  (41 - 76) | 60  (33 - 75) |
| CD1a  [%] | 84  (44 - 87) | 80  (42 - 90) | 73  (58 - 85) | 76  (65 - 83) | 74  (47 - 91) | 77  (48 - 91) |

**Table S4 Surface molecule expression on BAL fluid myeloid DCs (mDCs)**

Shown is the expression of various surface molecules on BAL fluid mDCs (median values, minimum – maximum) of the participants in the 3 study arms (Placebo, Fluticasone, Fluticasone/Salmeterol), at the time points A (prior to treatment) and B (directly after treatment). The expression was calculated as the percentage of marker-positive mDCs in most cases. In case of CD40, the expression was calculated using the Mean Fluorescence Index (MFI). *Abbreviation denotes:* Blood Dendritic Cell Antigen (BDCA). There were no significant differences between the time points in any parameter in any group.
